# Supplementary material for: Correlation among experience of person-centered maternity care, provision of care and women’s satisfaction: Cross sectional study in Colombo, Sri Lanka
Source: PLoS One. 2021 Apr 8;16(4):e0249265. doi: 10.1371/journal.pone.0249265 (PMC8031099; doi:10.1371/journal.pone.0249265)
Supplement: S2 Table — (DOCX) [file pone.0249265.s002.docx]

# S2 Table. PCMC scale and sub-scales

| **Subscales of PCMC tool** | **Number of items** | **Score (min-max) range** |
| --- | --- | --- |
| 1. **Dignity and respect** | 6 | 0-18 |
| 1. **Communication and autonomy** | 9 | 0-27 |
| 1. **Supportive Care** | 15 | 0-45 |
| **TOTAL** | 30 | 0-90 |
