# Supplementary material for: Correlation among experience of person-centered maternity care, provision of care and women’s satisfaction: Cross sectional study in Colombo, Sri Lanka
Source: PLoS One. 2021 Apr 8;16(4):e0249265. doi: 10.1371/journal.pone.0249265 (PMC8031099; doi:10.1371/journal.pone.0249265)
Supplement: S3 Table — (DOCX) [file pone.0249265.s003.docx]

# S3 Table. Study findings on the PCMC questionnaire

|  | **Mean**  **(95% CI)** | **SD** | **Min-max Range** | **Possible range** |
| --- | --- | --- | --- | --- |
| **Full PCMC Scale (30 items)** | 42.3  (41.3-43.4) | 10.7 | 14-82 | 0-90 |
| **Dignity & Respect (6 items)** | 10.3  (10.0-10.6) | 2.6 | 2-18 | 0-18 |
| **Communication & Autonomy (9 items)** | 9.3  (9.0-9.7) | 3.8 | 0-23 | 0-27 |
| **Supportive care (15 items)** | 22.7  (22.1-23.4) | 6.6 | 7-42 | 0-45 |
