# Supplementary material for: Correlation among experience of person-centered maternity care, provision of care and women’s satisfaction: Cross sectional study in Colombo, Sri Lanka
Source: PLoS One. 2021 Apr 8;16(4):e0249265. doi: 10.1371/journal.pone.0249265 (PMC8031099; doi:10.1371/journal.pone.0249265)
Supplement: S4 Table — (DOCX) [file pone.0249265.s004.docx]

# S4 Table. Frequency of each item on the PCMC scale

|  | **n** | **%** |
| --- | --- | --- |
| **Items under dignity and respect subscale** |  |  |
| Did the doctors, nurses, or other staff at the facility treat you with respect? | | |
| 0 No, none of them | 73 | 18.3 |
| 1 Yes, a few of them | 11 | 2.8 |
| 2 Yes, most of them | 62 | 15.5 |
| 3 Yes, all of them | 254 | 63.5 |
| Did the doctors, nurses, and other staff at the facility treat you in a friendly manner? | | |
| 0 No, none of them | 397 | 99.3 |
| 1 Yes, a few of them | 2 | 0.5 |
| 2 Yes, most of them | 1 | 0.3 |
| 3 Yes, all of them | 0 | 0.0 |
| Did you feel the doctors, nurses, or other health-care providers shouted at you, scolded, insulted, threatened, or talked to you rudely? | | |
| 0 No, none of them | 38 | 9.5 |
| 1 Yes, a few of them | 85 | 21.3 |
| 2 Yes, most of them | 163 | 40.8 |
| 3 Yes, all of them | 114 | 28.5 |
| Did you feel like you were treated roughly like pushed, beaten, slapped, pinched, physically restrained, or gagged? | | |
| 0 No, none of them | 12 | 3 |
| 1 Yes, a few of them | 159 | 39.8 |
| 2 Yes, most of them | 170 | 42.5 |
| 3 Yes, all of them | 59 | 14.8 |
| During examinations in the labour room, were you covered up? | | |
| 0 No, none of them | 49 | 12.3 |
| 1 Yes, a few of them | 226 | 56.5 |
| 2 Yes, most of them | 112 | 28 |
| 3 Yes, all of them | 13 | 3.3 |
| Do you feel like your health information was or will be kept confidential at this facility? | | |
| 0 No, none of them | 89 | 22.3 |
| 1 Yes, a few of them | 144 | 36 |
| 2 Yes, most of them | 142 | 35.5 |
| 3 Yes, all of them | 25 | 6.3 |
| **Items under Communication and Autonomy subscale** |  |  |
| During your time in the health facility did the doctors, nurses, or other health-care providers introduce themselves to you when they first came to see you? | | |
| 0 No, none of them | 138 | 34.5 |
| 1 Yes, a few of them | 178 | 44.5 |
| 2 Yes, most of them | 73 | 18.3 |
| 3 Yes, all of them | 11 | 2.8 |
| Did the doctors, nurses, or other health-care providers call you by your name? | | |
| 0 No, none of them | 171 | 42.8 |
| 1 Yes, a few of them | 97 | 24.3 |
| 2 Yes, most of them | 67 | 16.8 |
| 3 Yes, all of them | 65 | 16.3 |
| Did you feel like the doctors, nurses or other staff at the facility involved you in decisions about your care? | | |
| 0 No, none of them | 220 | 55 |
| 1 Yes, a few of them | 126 | 31.5 |
| 2 Yes, most of them | 50 | 12.5 |
| 3 Yes, all of them | 4 | 1 |
| During the delivery, do you feel like you were able to be in the position of your choice? | | |
| 0 No, none of them | 137 | 34.3 |
| 1 Yes, a few of them | 129 | 32.3 |
| 2 Yes, most of them | 119 | 29.8 |
| 3 Yes, all of them | 15 | 3.8 |
| Did the doctors, nurses, or other staff at the facility speak to you in a language you could understand? | | |
| 0 No, none of them | 14 | 3.5 |
| 1 Yes, a few of them | 67 | 16.8 |
| 2 Yes, most of them | 95 | 23.8 |
| 3 Yes, all of them | 224 | 56 |
| Did the doctors, nurses, or other staff at the facility ask your permission or consent before doing procedures on you? | | |
| 0 No, none of them | 228 | 57 |
| 1 Yes, a few of them | 133 | 33.3 |
| 2 Yes, most of them | 37 | 9.3 |
| 3 Yes, all of them | 2 | 0.5 |
| Did the doctors and nurses explain to you why they were doing examinations or procedures on you? | | |
| 0 No, none of them | 138 | 34.5 |
| 1 Yes, a few of them | 192 | 48 |
| 2 Yes, most of them | 64 | 16 |
| 3 Yes, all of them | 6 | 1.5 |
| Did the doctors and nurses explain to you why they were giving you any medicine? | | |
| 0 No, none of them | 343 | 85.8 |
| 1 Yes, a few of them | 50 | 12.5 |
| 2 Yes, most of them | 6 | 1.5 |
| 3 Yes, all of them | 1 | 0.3 |
| Did you feel you could ask the doctors, nurses, or other staff at the facility any questions you had? | | |
| 0 No, none of them | 120 | 30 |
| 1 Yes, a few of them | 125 | 31.3 |
| 2 Yes, most of them | 45 | 11.3 |
| 3 Yes, all of them | 110 | 27.5 |
| **Items under Supportive Care subscale** |  |  |
| How did you feel about the amount of time you waited? Would you say it was | | |
| 0 Very short | 93 | 23.3 |
| 1 Somewhat short | 204 | 51 |
| 2 Somewhat long | 90 | 22.5 |
| 3 Very long | 13 | 3.3 |
| Did the doctors and nurses at the facility talk to you about how you were feeling? | | |
| 0 No, none of them | 200 | 50 |
| 1 Yes, a few of them | 4 | 1 |
| 2 Yes, most of them | 7 | 1.8 |
| 3 Yes, all of them | 189 | 47.3 |
| Did the doctors, nurses, or other staff at the facility try to understand your anxieties? | | |
| 0 No, none of them | 244 | 61 |
| 1 Yes, a few of them | 5 | 1.3 |
| 2 Yes, most of them | 7 | 1.8 |
| 3 Yes, all of them | 144 | 36 |
| When you needed help, did you feel the doctors, nurses, or other staff at the facility paid attention? | | |
| 0 No, none of them | 44 | 11 |
| 1 Yes, a few of them | 188 | 47 |
| 2 Yes, most of them | 149 | 37.3 |
| 3 Yes, all of them | 19 | 4.8 |
| Do you feel the doctors or nurses did everything they could to help control your pain? | | |
| 0 No, none of them | 87 | 21.8 |
| 1 Yes, a few of them | 161 | 40.3 |
| 2 Yes, most of them | 125 | 31.3 |
| 3 Yes, all of them | 27 | 6.8 |
| Were you allowed to have someone you wanted (outside of staff at the facility, such as family or friends) to stay with you during labour? | | |
| 0 No, none of them | 15 | 3.8 |
| 1 Yes, a few of them | 51 | 12.8 |
| 2 Yes, most of them | 81 | 20.3 |
| 3 Yes, all of them | 253 | 63.2 |
| Were you allowed to have someone you wanted to stay with you during delivery? | | |
| 0 No, none of them | 2 | 0.5 |
| 1 Yes, a few of them | 16 | 4 |
| 2 Yes, most of them | 40 | 10 |
| 3 Yes, all of them | 342 | 85.5 |
| Did you feel the doctors, nurses, or other staff at the facility took the best care of you? | | |
| 0 No, none of them | 84 | 21 |
| 1 Yes, a few of them | 92 | 23 |
| 2 Yes, most of them | 126 | 31.5 |
| 3 Yes, all of them | 98 | 24.5 |
| Did you feel you could completely trust the doctors, nurses, or other staff at the facility with regards to your care? | | |
| 0 No, none of them | 22 | 5.5 |
| 1 Yes, a few of them | 143 | 35.8 |
| 2 Yes, most of them | 144 | 36 |
| 3 Yes, all of them | 91 | 22.8 |
| Do you think there were enough health staff in the facility to care for you? | | |
| 0 No, none of them | 39 | 9.8 |
| 1 Yes, a few of them | 189 | 47.3 |
| 2 Yes, most of them | 143 | 35.8 |
| 3 Yes, all of them | 29 | 7.2 |
| Thinking about the labour and postnatal wards, did you feel the health facility was crowded? | | |
| 0 No, none of them | 159 | 39.8 |
| 1 Yes, a few of them | 57 | 14.2 |
| 2 Yes, most of them | 69 | 17.3 |
| 3 Yes, all of them | 115 | 28.7 |
| Thinking about the wards, washrooms, and the general environment of the health facility, would you say the facility was very clean, clean, dirty, or very dirty? | | |
| 0 Very dirty | 19 | 4.8 |
| 1 Dirty | 116 | 29 |
| 2 Clean | 253 | 63.2 |
| 3 Very clean | 12 | 3 |
| Was there water in the facility? | | |
| 0 No, none of them | 86 | 21.5 |
| 1 Yes, a few of them | 54 | 13.5 |
| 2 Yes, most of them | 71 | 17.8 |
| 3 Yes, all of them | 189 | 47.3 |
| Was there electricity in the facility? | | |
| 0 No, none of them | 12 | 3 |
| 1 Yes, a few of them | 64 | 16 |
| 2 Yes, most of them | 121 | 30.3 |
| 3 Yes, all of them | 203 | 50.7 |
| In general, did you feel safe in the health facility? | | |
| 0 No, none of them | 25 | 6.3 |
| 1 Yes, a few of them | 126 | 31.5 |
| 2 Yes, most of them | 147 | 36.8 |
| 3 Yes, all of them | 102 | 25.5 |
