# Supplementary material for: Correlation among experience of person-centered maternity care, provision of care and women’s satisfaction: Cross sectional study in Colombo, Sri Lanka
Source: PLoS One. 2021 Apr 8;16(4):e0249265. doi: 10.1371/journal.pone.0249265 (PMC8031099; doi:10.1371/journal.pone.0249265)
Supplement: S5 Table — (DOCX) [file pone.0249265.s005.docx]

# S5 Table. Pearson correlation between the PCMC sub-scales and Bologna score

|  | **Full PCMC score** | **Dignity & Respect** | **Communication & Autonomy** | **Supportive Care** | **Bologna Score** |
| --- | --- | --- | --- | --- | --- |
| **Full PCMC score** | 1 |  |  |  |  |
| **Dignity & Respect** | 0.747 | 1 |  |  |  |
| **Communication & Autonomy** | 0.768 | 0.542 | 1 |  |  |
| **Supportive Care** | 0.901 | 0.516 | 0.471 | 1 |  |
| **Bologna Score** | 0.205 | 0.131 | 0.148 | 0.199 | 1 |

Abbreviation: PCMC = Person-centered maternity care
