# Supplementary material for: Correlation among experience of person-centered maternity care, provision of care and women’s satisfaction: Cross sectional study in Colombo, Sri Lanka
Source: PLoS One. 2021 Apr 8;16(4):e0249265. doi: 10.1371/journal.pone.0249265 (PMC8031099; doi:10.1371/journal.pone.0249265)
Supplement: S6 Table — (DOCX) [file pone.0249265.s006.docx]

# S6 Table. Spearman correlation between the PCMC sub-scales, Bologna score and Total satisfaction

|  | **Full PCMC score** | **Dignity & Respect** | **Communication & Autonomy** | **Supportive Care** | **Bologna Score** |
| --- | --- | --- | --- | --- | --- |
| **Full PCMC score** | 1 |  |  |  |  |
| **Dignity & Respect** | 0.710 | 1 |  |  |  |
| **Communication & Autonomy** | 0.734 | 0.500 | 1 |  |  |
| **Supportive Care** | 0.905 | 0.497 | 0.446 | 1 |  |
| **Bologna Score** | 0.205 | 0.132 | 0.157 | 0.205 | 1 |
| **Total Satisfaction** | 0.576 | 0.436 | 0.347 | 0.557 | 0.118 |

Abbreviation: PCMC = Person-centered maternity care.
