# Supplementary material for: Correlation among experience of person-centered maternity care, provision of care and women’s satisfaction: Cross sectional study in Colombo, Sri Lanka
Source: PLoS One. 2021 Apr 8;16(4):e0249265. doi: 10.1371/journal.pone.0249265 (PMC8031099; doi:10.1371/journal.pone.0249265)
Supplement: S7 Table — (DOCX) [file pone.0249265.s007.docx]

# S7 Table. Absolute frequency and percentage of satisfaction score dichotomised at the minimum satisfaction limit of 6

|  | **Satisfaction score**  **n(%)** | |
| --- | --- | --- |
|  | **Score ≥6**  **N=295** | **Score <6**  **N=105** |
| Age  < 18 years   - 1. ears   2. years   35-39 years  >40 years | 5 (1.7)  91 (30.9)  165 (55.9)  29 (9.8)  5 (1.7) | 1 (1.0)  42 (40.0)  51 (48.6)  11 (10.5)  0 |
| Number of pregnancies  1  2  ≥3 | 130 (44.1)  85 (28.8)  80 (27.1) | 51 (48.6)  24 (22.9)  30 (28.6) |
| Education  None or Primary  Secondary  Higher | 1 (0.3)  264 (89.5)  30 (10.2) | 0  99 (94.3)  6 (5.7) |
| Employed  No  Yes | 245 (83.1)  50 (17.0) | 89 (84.8)  16 (15.2) |
| Ethnicity  Burger  Muslim  Sinhalese  Tamil | 97 (32.9)  142 (48.1)  56 (15.0)  0 | 41 (39.1)  42 (40.0)  21 (20.0)  1 (0.9) |
| Risk factors (any)  No  Yes | 146 (49.5)  149 (50.5) | 47 (44.8)  58 (55.2) |
| Labour onset  Spontaneous  Induction | 193 (65.4)  102 (34.6) | 76 (72.4)  29 (27.6) |
| Mode of delivery  Vaginal spontaneous  Vaginal operative | 284 (96.3)  11 (3.7) | 104 (99.1)  1 (0.9) |
| Hour of delivery  Day (from 7 AM to 6 PM)  Night (from 7 PM to 6 AM) | 160 (54.2)  132 (44.8) | 54 (54.4)  51 (48.6) |
| Adverse outcomes  No  Yes | 261 (88.5)  34 (11.5) | 87 (82.9)  18 (17.1) |
| **Bologna score components** |  |  |
| Presence of a companion  No  Yes | 272 (92.2)  23 (7.8) | 90 (85.7)  15 (14.3) |
| Use of partograph  No  Yes | 27(9.2)  268 (90.8) | 6 (5.7)  99 (94.3) |
| Absence of stimulation to labor  No  Yes | 281 (95.2)  14 (4.8) | 100 (95.2)  5 (4.8) |
| Delivery in non-supine position  No  Yes | 75 (25.4)  220 (74.6) | 32 (30.5)  73 (69.5) |
| Skin-to-skin care  No  Yes | 170 (57.6)  125 (42.4) | 65 (61.9)  40 (38.1) |
