# Supplementary material for: Correlation among experience of person-centered maternity care, provision of care and women’s satisfaction: Cross sectional study in Colombo, Sri Lanka
Source: PLoS One. 2021 Apr 8;16(4):e0249265. doi: 10.1371/journal.pone.0249265 (PMC8031099; doi:10.1371/journal.pone.0249265)
Supplement: S8 Table — Absolute frequency and percentage of satisfaction score dichotomized at the median value of 7. (DOCX) [file pone.0249265.s008.docx]

**S8 Table. Sensitivity analysis. Absolute frequency and percentage of satisfaction score dichotomized at the median value of 7**

|  | Satisfaction score  n(%) | | P value |
| --- | --- | --- | --- |
|  | Score ≥7  N=250 | Score <7  N=150 |  |
| Age  < 18 years   - 1. ears   2. years   35-39 years  >40 years | 4 (1.6)  73 (29.2)  142 (56.8)  26 (10.4)  5 (2.0) | 2 (1.3)  60 (40.0)  74 (49.3)  14 (9.3)  0 | 0.230 |
| Number of pregnancies  1  2  ≥3 | 102 (40.8)  78 (31.2)  70 (28.0) | 79 (52.7)  31 (20.7)  40 (26.7) | 0.032 |
| Education  None or Primary  Secondary  Higher | 0  227 (90.8)  23 (9.2) | 1 (0.7)  136 (90.7)  13 (8.7) | 0.872 |
| Employed  No  Yes | 213 (85.2)  97 (14.8) | 121 (80.7)  29 (19.3) | 0.241 |
| Ethnicity  Burger  Muslim  Sinhalese  Tamil | 81 97 (32.4)  125 (50.0)  44 (17.6)  0 | 57 (38.0)  59 (39.3)  33 (22.0)  1 (0.7) | 0.127 |
| Risk factors (any)  No  Yes | 124 (49.6)  126 (50.4) | 69 (46.0)  81 (54.0) | 0.485 |
| Labour onset  Spontaneous  Induction | 166 (66.4)  84 (33.6) | 103 (68.7)  47 (31.3) | 0.640 |
| Mode of delivery  Vaginal spontaneous  Vaginal operative | 241 (96.4)  9 (3.6) | 147 (98.0)  3 (2.0) | 0.350 |
| Hour of delivery  Day (from 7 AM to 6 PM)  Night (from 7 PM to 6 AM) | 135 (54.0)  112 (44.8) | 79 (52.7)  71 (47.3) | 0.700 |
| Adverse outcomes  No  Yes | 223 (89.2)  27 (10.8) | 125 (83.3)  25 (16.7) | 0.095 |
| **Bologna score components** |  |  |  |
| Presence of a companion  No  Yes | 231 (92.4)  19 (7.6) | 131 (87.3)  19 (12.7) | 0.099 |
| Use of partograph  No  Yes | 22 (8.8)  228 (91.2) | 11 (2.8)  139 (92.7) | 0.603 |
| Absence of stimulation to labor  No  Yes | 237 (94.8)  13 (5.2) | 144 (96.0)  6 (4.0) | 0.581 |
| Delivery in non-supine position  No  Yes | 58 (23.2)  192 (76.8) | 49 (32.7)  101 (67.3) | 0.039 |
| Skin-to-skin care  No  Yes | 146 (58.4)  104 (41.6) | 89 (59.3)  61 (40.7) | 0.854 |
