# Supplementary material for: Correlation among experience of person-centered maternity care, provision of care and women’s satisfaction: Cross sectional study in Colombo, Sri Lanka
Source: PLoS One. 2021 Apr 8;16(4):e0249265. doi: 10.1371/journal.pone.0249265 (PMC8031099; doi:10.1371/journal.pone.0249265)
Supplement: Attachment — Submitted filename: Commenti Paper Rishard ML0511_BA1111IM.docx [file pone.0249265.s010.docx]

CC: esacks@jhsph.edu

PONE-D-20-18688
Correlation among experience of person-centered maternity care, provision of care and women’s satisfaction: cross sectional study in Colombo, Sri Lanka
PLOS ONE

Dear Dr. Rishard,

Thank you for submitting your manuscript to PLOS ONE. After careful consideration, we feel that it has merit but does not fully meet PLOS ONE’s publication criteria as it currently stands. Therefore, we invite you to submit a revised version of the manuscript that addresses the points raised during the review process.

Please submit your revised manuscript by Oct 29 2020 11:59PM. If you will need more time than this to complete your revisions, please reply to this message or contact the journal office at plosone@plos.org. When you're ready to submit your revision, log on to https://www.editorialmanager.com/pone/ and select the 'Submissions Needing Revision' folder to locate your manuscript file.

Please include the following items when submitting your revised manuscript:

- A rebuttal letter that responds to each point raised by the academic editor and reviewer(s). You should upload this letter as a separate file labeled 'Response to Reviewers'.
- A marked-up copy of your manuscript that highlights changes made to the original version. You should upload this as a separate file labeled 'Revised Manuscript with Track Changes'.
- An unmarked version of your revised paper without tracked changes. You should upload this as a separate file labeled 'Manuscript'.

If you would like to make changes to your financial disclosure, please include your updated statement in your cover letter. Guidelines for resubmitting your figure files are available below the reviewer comments at the end of this letter.

If applicable, we recommend that you deposit your laboratory protocols in protocols.io to enhance the reproducibility of your results. Protocols.io assigns your protocol its own identifier (DOI) so that it can be cited independently in the future. For instructions see: [http://journals.plos.org/plosone/s/submission-guidelines#loc-laboratory-protocols](https://posta.um.fvg.it/owa/redir.aspx?C=dpmHsBA_-qYNpSKQS0vlVTpLB9xV-OSi7pEFA-qTfJrM3BIxE1vYCA..&URL=http%253a%252f%252fjournals.plos.org%252fplosone%252fs%252fsubmission-guidelines%2523loc-laboratory-protocols)

We look forward to receiving your revised manuscript.

Kind regards,

Emma Sacks
Academic Editor
PLOS ONE

Journal Requirements:

When submitting your revision, we need you to address these additional requirements.

1. Please ensure that your manuscript meets PLOS ONE's style requirements, including those for file naming. The PLOS ONE style templates can be found at

https://journals.plos.org/plosone/s/file?id=wjVg/PLOSOne_formatting_sample_main_body.pdf and

https://journals.plos.org/plosone/s/file?id=ba62/PLOSOne_formatting_sample_title_authors_affiliations.pdf

 content.

***The format of the manuscript has been revised following the above guidelines

2. Please provide additional details regarding participant consent. In the ethics statement in the Methods and online submission information, please ensure that you have specified (1) whether consent was informed and (2) what type you obtained (for instance, written or verbal, and if verbal, how it was documented and witnessed). If your study included minors, state whether you obtained consent from parents or guardians. If the need for consent was waived by the ethics committee, please include this information.

 ***Thank you for the comment. We have provided additional details in the paper. We obtained ethical approval from the ethics review committee and permission from the director of De Soysa Hospital for women before conducting the interviews. We provided the informed consent form in Tamil, English and Singhala. Details regarding the research were provided by a research assistant who had prior experience in research and a degree in biomedical science. Informed written consent was taken from participants. In case of minors informed assent and consent from guardians / parents were obtained.

3. Please include captions for your Supporting Information files at the end of your manuscript, and update any in-text citations to match accordingly. Please see our Supporting Information guidelines for more information: http://journals.plos.org/plosone/s/supporting-information.

***The Supporting Information files and their citation in the manuscript have been revised following the above guidelines

Additional Editor Comments (if provided):

BEFORE SUBMITTING A REVISION, PLEASE ADD PAGE AND LINE NUMBERS. IT IS VERY DIFFICULT FOR REVIEWERS TO GIVE COMMENTS WITHOUT THESE.
***Thank you for the suggestion. We revised the format of the manuscript

While this is a critically important topic, and clearly understudied in Sri Lanka, this paper needs significantly more analytic work. The analyses are very basic and present only the totals, despite the methods indicating the ability to assess various correlations between demographics and practices, and between the PCMC, Bologna, and Likert scales. The results presented here are cursory. Detailed results should be in the paper, not supplementary appendices.

***Thank you for your comment, which gave us the opportunity to improve the analysis and to report relevant results in the main text. We have now moved some of the key results from the supplementary appendices to the manuscript, such as score distributions. Moreover, bivariate and multivariate analyses were performed and reported in the manuscript and a sensitivity analysis was conducted.

This paper requires more editing for grammar (even in the first paragraph, there are mistakes) and spelling (including Caesarean etc).
Please spell out acronyms, including PCMC, IUGR etc, at first use and in tables.

***Thank you for the comment. The grammar and spelling were revised and edited by a native English speaker.

Please include in limitations that *not including women with psychiatric illnesses may introduce bias, as these women may be more likely to experience mistreatment*.

***Thank you for the comment. The limitation suggested has been included in line 3 of the last paragraph of the discussion.

Please include information about how women below age 18 were consented/assented.

***Thank you for this observation, women below age 18 were consented /assented.

Please include more specifics in the methods - how were women recruited? what is an "established individual patient database" = are these hospital records?
***Thank you for your comment. We have clarified the procedure related to the collection on women characteristics and outcomes in the lines 148-150. This data collection process has been in practice since July 2015.

In relation to the other indicators, eligible mothers were identified using the birth registry: all the consecutive deliveries were approached. All eligible women who consented to participate in the study were interviewed. Deliveries that took place during daytime, nights and weekends were included.

Please include more details about the sample size calculation.

***Thank you for the comment which gave us the opportunity to better clarify the methods and to include specific details as suggested.

Please explain how it was ensured that women understood that research assistants were not hospital staff (which may have led to desirability bias)

***Desirability bias was minimised by having an interviewer who is not a staff member. Both the interviewers’ outfit/uniform and the identification card clearly allowed them to be identified as non-staff members. Beside this, interviewers introduced themself and their role. Furthermore, they clarified that interviews were anonymous. Interviews were conducted in a separate area with privacy.

Why were indicators chosen like "non supine" rather than "women's preferred birthing positions were respected"? Wouldn't that be more person-centered?

***Thank you for your observation. We very much agree with you in principle, but we had to adhere to a predefined scoring system. In fact, we followed the definitions provided by the Bologna Score System, which uses delivery in non-supine as the indicator to measure this practice. Women’s preferences in birthing positions will be encouraged during the future interventions to improve quality of care.

When referring to "risk factors" please clarify "medical risk factors" (to differentiate from social risk)

***Thank you for the suggestion. The text was revised accordingly.

"Near miss" is vague for a medical event - can you include the medical conditions which caused these severe morbidities?

***Thank you for the comment. The definition of “maternal near miss” was added in the notes of table 1. The definition was agreed locally. It is based on the recommendation of the WHO Manual “Evaluating the quality of care for severe pregnancy complications: the WHO near-miss approach for maternal health.” <https://apps.who.int/iris/bitstream/handle/10665/44692/9789241502221_eng.pdf;jsessionid=4BB1351F7F82F8B755AA9220A11279B4?sequence=1>

Figure 1 is very basic and could be strengthened by including bars comparing average PCMC scores for various subpopulations (the 3 sub categories can easily be shown in a stacked bar as part of the total)

***Thank you for the comment. We improved the figure, which at the moment is figure 2, showing each component of the PCMC score normalised to 100 to allow a better comparison among full score and subscores. We also inserted another figure (which in the current manuscript is Fig 1) where we reported the distribution of PCMC, Bologna score and women’s satisfaction Likert scale

How did 99% of women think they were treated poorly by health professions but 63% thought they were treated with respect - is this not measuring the same thing?

*** Thank you for your observation. The PCMC questionnaire includes the following two questions: 1) Did the doctors, nurses, or other staff at the facility treat you with respect? 2) Did the doctors, nurses, and other staff at the facility treat you in a friendly manner?

These questions focus on related yet different aspects of respectful care. Results support the hypothesis that not being treated in a friendly manner is not perceived to be a discriminating component for respectful care.

The percent of women delivering without a companion should probably be shown with the denominator of those who wanted to have a companion, as that would be a better proxy for respectful care.

*** Thank you for your suggestion. We decided to show percentages of reasons for absence of a companion at the time of birth with the number of women without a companion as denominator to give emphasis to the impact of socio-cultural context on women’s preferences (please see Table 2).

What is the definition of "supportive care"? How was "maternal outcome" assessed?
***Thank you for the question. The PCMC questionnaire includes three subdomains, the specification of these has been inserted in table S4. One of these is called “Supportive care”. This subdomain includes 15 items which measure supportive care and are related to the waiting time, the pain control, the companion during delivery, the hospital environment. We provide hereby a reference “Afulani PA, Phillips B, Aborigo RA, Moyer CA. Person-centred maternity care in low-income and middle-income countries: analysis of data from Kenya, Ghana, and India. Lancet Glob Health. 2019 Jan;7(1):e96-e109. doi: 10.1016/S2214-109X(18)30403-0.”

For maternal outcomes we intended maternal health outcomes. we have clarified this better in the text

The discussion section could use more detail:
If many women had low PCMC scores but reported high satisfaction, does that indicate that expectations are low? Can this be unpacked - what is the literature on this about social forces that contribute to these expectations?

***Thank you for your comment, which gave us the opportunity to add an explanation and additional references in the discussion section.

Although the PCMC tool focuses on the experience of care and includes different dimensions such as communication, respect, dignity, and emotional support, which are highlighted as key dimensions also in the World Health Organization (WHO) quality of care framework for maternal and newborn health (Tunçalp et al, BJOG 2015), women satisfaction may be depended on many other factors. We report below what literature reports as key aspects that contribute to these expectations

- Women construct their birth experience differently. Views are directed by personal beliefs and values (Martin CH, Fleming V. The birth satisfaction scale. Int J Health Care Qual Assur. 2011;24(2):124-35. doi: 10.1108/09526861111105086. PMID: 21456488)
- Ethnicity, religion, facility location have all been shown to influence women’s satisfaction. (Srivastava, A., Avan, B.I., Rajbangshi, P. et al. Determinants of women’s satisfaction with maternal health care: a review of literature from developing countries. BMC Pregnancy Childbirth 15, 97 (2015). <https://doi.org/10.1186/s12884-015-0525-0>
- It has been described that women tend to normalize the disrespectful care when they experience good outcomes (Freedman LP, Kruk ME. Disrespect and abuse of women in childbirth: challenging the global quality and accountability agendas. Lancet. 2014;384:e42–4 and Bowser D, Hill K. Exploring evidence and abuse in facility-based childbirth: report of a landscape analysis. Washington DC: USAID-TRAction Project; 2010. Available from: http://wwwtractionprojectorg/sites/default/files/ Respectful_Care_at_Birth_9-20-101_Final.pdf)

Our findings of high satisfaction rate could be due to perception of satisfaction that women generally feel after the arrival of their newborns, in a context where this is the priority value.

If women delivered by a nurse had better PCMC scores than those delivered by a midwife, what is the hypothesis about how nurses vs midwives are trained?

***Thank you for the comment. We have further checked this variable. In real practice nurses and midwives work as members. Mothers usually receive care from both cadres at different times. In addition, mothers may find difficult to identify correctly nurses from midwife. For these reasons, we decided to exclude the variable related to the person who delivered from the analysis.

Why might practices be different vs day than night? Are there different staffing levels or cadres available?
***Thank you for the comment. In many settings staffing levels are lower at night. In the study setting staff allocation at night is less. Onsite consultant is also available only during daytime, while during nights is “on call”.

Figures 3A, B, and C are too blurry to read.
*** Thank you for the comment. The figures were revised.


Reviewers' comments:

Reviewer's Responses to Questions

**Comments to the Author**

1. Is the manuscript technically sound, and do the data support the conclusions?

The manuscript must describe a technically sound piece of scientific research with data that supports the conclusions. Experiments must have been conducted rigorously, with appropriate controls, replication, and sample sizes. The conclusions must be drawn appropriately based on the data presented.

Reviewer #1: Partly

Reviewer #2: Yes

2. Has the statistical analysis been performed appropriately and rigorously?

Reviewer #1: Yes

Reviewer #2: Yes

3. Have the authors made all data underlying the findings in their manuscript fully available?

The [PLOS Data policy](https://posta.um.fvg.it/owa/redir.aspx?C=bNwi-Zw_ByVb-gZxjz_4UByVxKYfNm-xp3mLqwqV1QTM3BIxE1vYCA..&URL=http%253a%252f%252fwww.plosone.org%252fstatic%252fpolicies.action%2523sharing) requires authors to make all data underlying the findings described in their manuscript fully available without restriction, with rare exception (please refer to the Data Availability Statement in the manuscript PDF file). The data should be provided as part of the manuscript or its supporting information, or deposited to a public repository. For example, in addition to summary statistics, the data points behind means, medians and variance measures should be available. If there are restrictions on publicly sharing data—e.g. participant privacy or use of data from a third party—those must be specified.

Reviewer #1: No

Reviewer #2: Yes

4. Is the manuscript presented in an intelligible fashion and written in standard English?

PLOS ONE does not copyedit accepted manuscripts, so the language in submitted articles must be clear, correct, and unambiguous. Any typographical or grammatical errors should be corrected at revision, so please note any specific errors here.

Reviewer #1: Yes

Reviewer #2: No

5. Review Comments to the Author

Please use the space provided to explain your answers to the questions above. You may also include additional comments for the author, including concerns about dual publication, research ethics, or publication ethics. (Please upload your review as an attachment if it exceeds 20,000 characters)

Reviewer #1: Overall, I think this is an area of needed research, especially understanding the intersecting relationships between person-centered maternity care, quality of clinical care, and women’s evaluations of the care experience. However, it appears that this study may need additional analyses, because I am not sure we are getting the full picture.

***Thank you for your appreciation.

1. According to Chalmers & Porter (2003), the Bologna score quantifies “the extent to which labors have been managed as if normal as opposed to complicated.” Given that 51.8% of women had at least one risk factor and that 13.0% of women had at least one adverse outcome, please justify the use of this scale for this purpose among this sample.

***Thank you for your comment. Chalmers & Porter (2003) stated that this tool attempts to quantify to which extend the labour is managed normally as opposed to complicated labour. In terms of labour, all women included in our study had a physiological labour.

To our knowledge having a medical risk factor (such as diabetes or hypertension) or having, after physiological labour, an adverse health outcome is not a criterion of exclusion for using the Bologna Score.

This tool was designed to use to study the behaviours and practices towards normal labour care within the maternity services. Further this tool can be used to assess and compare the childbirth support process and practice of evidence-based care throughout the world.

(BIRTH 35:4 December 2008 321 Care in Labor: A Swedish Survey Using the Bologna Score Ann-Kristin Sandin-Bojo ̈, RN, RM, PhD, and Linda J. Kvist, RN, RM, PhD)

2. Satisfaction scores are only informative to the degree that they indicate what women are satisfied with. What is the question used for “total satisfaction”? The frame of the question will help give context to what women were actually evaluating (e.g. was it satisfaction with care, the experience of childbirth, etc.?).
*** The question was phrased as follows “What is your overall satisfaction with care received, on a scale 1 (minimum )to 10 (maximum)?

Several other studies used this direct simple question to assess the overall degree of satisfaction of mothers. Many studies also use this phrasing to assess service users satisfaction with care received.

References:

1. Lazzerini M, Mariani I, Semenzato C, Valente EP. Association between maternal satisfaction and other indicators of quality of care at childbirth: a cross-sectional study based on the WHO standards. BMJ Open. 2020 Sep 14;10(9):e037063. doi: 10.1136/bmjopen-2020-037063. PMID: 32928854; PMCID: PMC7490935.
2. Conesa Ferrer MB, Canteras Jordana M, Ballesteros Meseguer C, et al. Comparative study analysing women's childbirth satisfaction and obstetric outcomes across two different models of maternity care. BMJ Open 2016;6:e011362. 10.1136/bmjopen-2016-011362.
3. Alfaro Blazquez R, Corchon S, Ferrer Ferrandiz E. Validity of instruments for measuring the satisfaction of a woman and her partner with care received during labour and childbirth: systematic review. Midwifery 2017;55:103–12. 10.1016/j.midw.2017.09.014.

3. I think this study could benefit from further analysis.

a. First, descriptions and results of multivariate analysis could be better described and presented (e.g. what were the variables included in the final multivariate model? What are the estimates associated with PCMC or Bologna score in the multivariate models).

***Thank you for your suggestion, which give us the opportunity to improve the paper we added a table “Table 2. Two- and multi-ways ANOVA” in the manuscript with the results of the analysis as suggested.

b. Though satisfaction was evaluated on a Likert scale, the multivariate analysis used logistic regression, splitting satisfaction into 6 and above versus under 6. It seems that linear regression seems more appropriate, assessing the incremental impact of the PCMC or Bologna scales.
***Thank you for your comment. We opted for a logistic regression model based on the fact that the assumptions for a linear regression models did not hold (eg, normality) therefore both linear regression estimates and relative statistic tests could be biased. The cut off at 6 was taken as the minimum satisfaction limit. Moreover, as described in the method section, a sensitivity analysis was performed dichotomizing satisfaction Likert scale at its median value. Results of the sensitivity analysis are shown in S8 Table and reported in the result section in the paragraph “Univariate and multivariate analyses” (lines 367-371).

c. Furthermore, the indicators included in the Bologna scale may not carry equal weight for women’s perceptions of care, especially because satisfaction may be based on women’s expectations and conditional on their social context. For instance, because induction of labor is commonly practiced and may be expected, it might not negatively impinge on women’s satisfaction. Perhaps assessments of individual indicators with women’s satisfaction might reveal the extent to which certain practices are correlated with satisfaction in this context.

***Thank you for your comment. We fully agree. We revised the paper and assessed the association between each component of the Bologna score with women’s satisfaction including them in the bivariate and multivariate analysis.

d. One of the measures in the Bologna score was the presence of a labour companion, however many of the qualitative results (Supplement, Table 6) indicate that many women did not want a labor companion. In this case, the corresponding indicator within the Bologna scale would not represent better clinical care (for example, providers respecting a woman's decision to not include a companion would represent higher quality care). This should, in some way, be considered in your handling of the Bologna scale (and especially accounted for in your multivariate model).
*** Thank you for your suggestion which gave us the opportunity to better clarify this point in the paragraph “Provision of care” in the result section. We have refined our analysis, based also on the recommendation of another review, in order to report these data in more detail (Figure 3, table 2, and text lines 316-319).

Smaller issues:
For figure 1, scores are not easily comparable since they use different scales. It might be more helpful to display scores as percentages, so that they will be presented on the same scale.

***Thank you for your suggestion. To allow an easy comparison across the different PCMC domains, rescaled scores were calculated as the fraction of the total possible score on each domain and normalised to 100 (in line with what done by Afulani, Lancet global health 2020).

Do you have information about what factors are associated with induction of labour, either qualitative or quantitative? Is it regular practice based on longer labours? Is it based on certain criteria of women’s conditions? Did they tend to be at night, etc.?
*** Thank you for your suggestion. We strongly believe that it would be beneficial for our setting to address the Induction of labour (IOL) in a separate paper, which indeed has already been planned. We are in the process of conducting a comprehensive audit of IOL practices over 4 years, to identify what factors would have led to high rates of IOL. This separate paper shall be submitted soon.

In the Supplementary Files, Table 5a-c, categorizes each measure into 3 groups. What is the reasoning behind these specific groupings? (For example, why is the first category for PCMC 0-58?)

***Thank you for the comment. Based on referee’s comments we decided to represent the table previously listed as Table 5a-c of Supplementary Files differently, and now it became figure 1. In this Figure 1 we reported the distributions of the scores through histograms and estimated normal distributions for subpopulations which resulted statistically significant in the bivariate analysis.

Reviewer #2: This is an interesting article on an important and timely topic. Methods are clearly described, the authors used reliable study measures and are transparent about their protocols and procedures.

***Thank you for your appreciation.

Overall the literature review is a bit thin – could use more of a rationale for why they chose to use a measure of satisfaction when for some time now researchers have understood satisfaction to be a poor discriminator of quality, in some LMICS, that their expectations for a low level of supportive care and the relief of having a live baby often leads to higher satisfaction scores even when they or observers report objective evidence of mistreatment. The discussion would also be enhanced with a bit more in depth exploration of some of the findings, as indicated in my notes below. In particular a discussion about the findings on quality of care as relates to global evidence on the overuse of interventions is missing – the authors limit their discussion to a small section on the components of the Bologna score without exploring the disconnect between overuse of interventions and patient satisfaction.
***Thank you for your comment and suggestion which gave us the opportunity to refine the literature and the discussion.

We report hereby the references added:

- Ghana

Dzomeku, V.M., Boamah Mensah, A.B., Nakua, E.K., Agbadi P, Lori JR, Donkor P. “I wouldn’t have hit you, but you would have killed your baby:” exploring midwives’ perspectives on disrespect and abusive Care in Ghana. BMC Pregnancy Childbirth 20, 15 (2020).

- Iran

Hajizadeh, K., Vaezi, M., Meedya, S., Charandabi ASM, Mirghafourvand M. Prevalence and predictors of perceived disrespectful maternity care in postpartum Iranian women: a cross-sectional study. BMC Pregnancy Childbirth 20, 463 (2020). https://doi.org/10.1186/s12884-020-03124-2

- South African

Lappeman, M., Swartz, L. Rethinking obstetric violence and the “neglect of neglect”: the silence of a labour ward milieu in a South African district hospital. BMC Int Health Hum Rights 19, 30 (2019). https://doi.org/10.1186/s12914-019-0218-2

Sharma, G., Penn-Kekana, L., Halder, K. Filippi V. An investigation into mistreatment of women during labour and childbirth in maternity care facilities in Uttar Pradesh, India: a mixed methods study. Reprod Health 16, 7 (2019). https://doi.org/10.1186/s12978-019-0668-y

- Ethiopia

Kassa, Z.Y., Husen, S. Disrespectful and abusive behavior during childbirth and maternity care in Ethiopia: a systematic review and meta-analysis. BMC Res Notes 12, 83 (2019). https://doi.org/10.1186/s13104-019-4118-2

Gebremichael, M.W., Worku, A., Medhanyie, Edin AAK, Berhane Y. Women suffer more from disrespectful and abusive care than from the labour pain itself: a qualitative study from Women’s perspective. BMC Pregnancy Childbirth 18, 392 (2018). https://doi.org/10.1186/s12884-018-2026-4

Ukke GG, Gurara MK, Boynito WG. Disrespect and abuse of women during childbirth in public health facilities in Arba Minch town, south Ethiopia - a cross-sectional study. PLoS One. 2019 Apr 29;14(4):e0205545. doi: 10.1371/journal.pone.0205545. PMID: 31034534; PMCID: PMC6488058.

- Tanzania

Kruk ME, Kujawski S, Mbaruku G, Ramsey K, Moyo W, Freedman LP. Disrespectful and abusive treatment during facility delivery in Tanzania: a facility and community survey. Health Policy Plan. 2018 Jan 1;33(1):e26-e33. doi: 10.1093/heapol/czu079. PMID: 29304252.

- Brazil

Lansky S, Souza KV, Peixoto ERM, Oliveira BJ, Diniz CSG, Vieira NF, et al. Obstetric violence: influences of the Senses of Birth exhibition in pregnant women childbirth experience. Cien Saude Colet. 2019 Aug 5;24(8):2811-2824. Portuguese, English. doi: 10.1590/1413-81232018248.30102017. PMID: 31389530.

- Nigeria

Ishola F, Owolabi O, Filippi V. Disrespect and abuse of women during childbirth in Nigeria: A systematic review. PLoS One. 2017 Mar 21;12(3):e0174084. doi: 10.1371/journal.pone.0174084. PMID: 28323860; PMCID: PMC5360318.

- Myanmar

Maung, T.M., Show, K.L., Mon, N.O. et al. A qualitative study on acceptability of the mistreatment of women during childbirth in Myanmar. Reprod Health 17, 56 (2020). <https://doi.org/10.1186/s12978-020-0907-2>

Additional references:

Bohren MA, Mehrtash H, Fawole B, Maung TM, Balde MD, Maya E, et al. How women are treated during facility-based childbirth in four countries: a cross-sectional study with labour observations and community-based surveys. Lancet. 2019 Nov 9;394(10210):1750-1763. doi: 10.1016/S0140-6736(19)31992-0. Epub 2019 Oct 8. PMID: 31604660; PMCID: PMC6853169.

Brizuela V, Leslie HH, Sharma J, Langer A, Tunçalp Ö. Measuring quality of care for all women and newborns: how do we know if we are doing it right? A review of facility assessment tools. Lancet Glob Health. 2019;7:e624–e632

The manuscript is mostly well organized and written in acceptable English but there are issues with syntax, missing plurals, tense, and typographical errors throughout – Since PLOS does not copyedit before publishing, I strongly recommend the authors arrange for copyediting by a native English speaker who is a good editor before resubmission.

***Thank you for your suggestion. The manuscript was revised and edited by a native speaker

1. Some examples of English language errors:
• Notably, in Sri Lanka the maternal mortality rate had a major declined
over the last sixty years - it was 1694/100,000 in 1947 - to reach one of the lowest rate_ in the South Asian Region, despite Sri Lanka being a lower middle-income country [1]
• These remarkable achievements have been reached on the back of consistent commitment_ toward health and health-related policies, including as critical aspects (of?/as?) the provision of free of charge education and free of charge health services [3,4].
***The manuscript was revised and edited.

• For example, despite WHO explicitly recommends labor companionship as a low-cost intervention to improve outcomes of labor [16], and despite Sri Lankan government has explicitly included this in a national policy [17], a recent survey highlighted that nearly 60% of consultant obstetricians did not allow labour companions in their wards [18].

***The manuscript was revised and edited.

• Women who underwent a caesarian section, or with an age outside the inclusion criteria, or with major psychiatric illnesses, or hospitalized in intensive care unit, or refusing consent, were excluded.

*** The manuscript was revised and edited.

• On the other side, the PCMC score significantly changed in different ethnic group, in women with more pregnancies, and by type of professionals that assisted the delivery.

*** The manuscript was revised and edited.

2. There are now several studies exploring mistreatment and abuse of women during pregnancy globally – please specify if you are referring to South Asian studies….
“Although few studies have explored the area of mistreatment and abuse of women during pregnancy, existing qualitative reports suggest a tendency for discriminatory behavior (such as verbal, emotional and even sexual abuse) and a diffuse normalization of disrespectful and abusive treatment of female patients [19,20”
***Thank you for your comment. Indeed we were referring to south Asia, we specified it accordingly in the manuscript.

3. Please specify what type of ‘training” the researcher received:
“The questionnaire was administered in the immediate post-natal period, before discharge, by an independent trained researcher. “
***Thank you for the comment. The trained researcher had prior experience in patient interviewing. She was trained directly by the principal investigator on all standard operating procedures (SOP), which included; women’s eligibility criteria, how to approach mothers, privacy, data quality checks etc. She was supervised high-intensity in the field for a week in which she improved the skills in taking informed consent, interviewing, and recording data. Once the primary investigator felt that all queries were solved, she started data collection.

4. Re the discussion about the use of partograph as an indicator of quality via the Bologna Score: The WHO no longer recommends the use of partograph as a measure of quality: See these articles by their team:
Bonet M, Oladapo OT, Souza JP, Gulmezoglu AM. Diagnostic accuracy of the partograph alert and action lines to predict adverse birth outcomes: a systematic review. BJOG 2019;126:1524–1533.
Souza JP, Oladapo OT, Fawole B, Mugerwa K, Reis R, Barbosa-Junior F, Oliveira-Ciabati L, Alves D, G€ulmezoglu AM. Cervicaldilatation over time is a poor predictor of severe adverse birth outcomes: a diagnostic accuracy study. BJOG 2018;125:991–1000.
Please discuss the more current recommendations for monitoring, interpretation, and management of labour progress in light of your findings.

*** We have revised literature and we got in touch with WHO staff at head quarter.

- The paper that you refer to (Bonet M, Oladapo OT, Souza JP, Gulmezoglu AM. Diagnostic accuracy of the partograph alert and action lines to predict adverse birth outcomes: a systematic review. BJOG 2019;126:1524–1533.) assessed one specific parameter of the partograph, and concluded that “This systematic review does not support the use of the cervical dilatation over time (at a threshold of 1 cm/h during active first stage) to identify women at risk of adverse birth outcomes.” However, authors suggest that care providers should continue to use other partograph parameters to monitor the well‐being of the woman and her baby, and identify risks for adverse birth outcomes and highlight the need of new tools to improve birth outcomes and reduce of unnecessary interventions during labour. The WHO recommendation is in line with this <https://extranet.who.int/rhl/topics/preconception-pregnancy-childbirth-and-postpartum-care/care-during-childbirth/care-during-labour-1st-stage/who-recommendation-progress-first-stage-labour-diagnostic-test-accuracy-1%E2%80%93cm/hour-cervical>
- We could not retrieve any formal statement from WHO where the use of partogram is NOT recommended.
- Sri Lankan guidelines for maternal care has emphasized the use of partogram <http://fhb.health.gov.lk/index.php/en/technical-units/maternal-care-unit>

5. Please justify the rationale for recoding the Likert scale for satisfaction into a binary especially in light of the subtleties in using satisfaction as a measure of quality of experience:
“Women satisfaction was analyzed as a binary outcome (Likert scale equal or more than 6 versus Likert scale less than 6) and the odds ratio (OR) of each predictor on it was calculated through bivariate logistic regression.”

***Thank you for your comment. We opted for a logistic regression model due to a deviation from the assumptions of linear regression models (ie, normality) therefore both linear regression estimates and relative statistic tests could be biased. The cut off at 6 was taken as the minimum satisfaction limit. Moreover, as described in the method section, a sensitivity analysis was performed dichotomizing satisfaction Likert scale at its median value. Results of the sensitivity analysis are shown in S8 Table and reported in the result section in the paragraph “Univariate and multivariate analyses” (lines 367-371).


6. Please specify how the women were “ involved in the study by providing their views on the quality of care received.” Did they participate in survey development? Pilot test? Content Validate the measures?

***Thank you for the comment. Women participated in the tool development and validation.

The tool has been validated in India and in other low middle-income countries where women had participated in the validation process. In our study, we included women in translation and adaptation process.

Four women Tamil speaking and four Sinhala speaking women were included in the expert committee, they examined the concordance with the original version. Two rounds of modifications were conducted and inadequate expressions/concepts of the translation, as well as any discrepancies, were corrected.

Further, 10 women who recently delivered, belonging to all age groups and from different socioeconomic backgrounds, were involved in pilot testing and content validity to check the understandability, clarity and acceptability of each tool separately.

Series of focus group discussion have been done to obtain women’s views on quality of care and that will be discussed in a different paper.

7. Points that need more in depth Discussion:
• Despite the following interesting finding: “Nearly two thirds (61.7%) were assisted by a nurse, one third (33.7%) by a midwife, and only a minority by a doctor.”there is almost no discussion about the differential effects of the type of provider on the quality of care (aside from noting women reported more respect by nurses than midwives) , nor explanation of potential reasons for these differences. This is important to unpack especially in light of global evidence that suggests that midwives provide more respectful care. Please also add some information in the background about the organization of care in Sri Lanka, the respective roles of providers, and describe the caseload vs service based models available.

***Based on comments from other referees we realized that this variable may not be accurate. Mothers are most often assisted by a team of people. They may have difficulties to distinguishing between a midwife and a nurse. For these reasons we have excluded this variable from the analysis.

• The mean PCMC score was significantly higher in Sinhalese women compared to Muslim (mean difference: 3.3; p=0.041) and to Tamil (mean difference: 3.8; p=0.049). S
This sentence and finding also deserves more attention in the discussion – please acknowledge this ethnic disparities in PCMC and address any implied or known cultural racism and bias that exists within the socio political climate, and contributes to these findings. This is not unlike other jurisdictions where marginalized populations experience more mistreatment (See Vedam et al. 2019, Giving Voice to Mothers, Reproductive Health).
***Thank you for your comments. We agree with your observation. We added this additional point and references in the discussion section.

8. Given the high rates of different types of mistreatment and violations of human rights reported, the emphasis in the following sentence appears misplaced. I suggest that the clause should begin with less than two thirds rather than nevertheless, and there should be some discussion about why this type of behavior was acceptable to those in the two thirds portion of the data.
“Notably, the majority of women (99.3%) reported to have been treated with an unfriendly manner by health professionals, nevertheless about two thirds (63.5%) thought that medical staff treated them with respect.”

***Thanks for this comment. In phrasing these results we did not want to sound judgmental. We have revised the sentence accordingly.

Treating patients in a friendly manner is a psychosocial skill that health professionals should possess, however a metanalysis pointed out the many underlying causes at different levels (individual, hospital, national level) which can affect these behaviours (*Mannava P, Durrant K, Fisher J, Chersich M, Luchters S. Attitudes and behaviours of maternal health care providers in interactions with clients: a systematic review. Global Health. 2015 Aug 15;11:36*).

9. Please take this opportunity discuss the following findings in light of global health human rights standards rather than deflecting this to a mandate for future study or simply development of courses to “promote PCMC”:
“Overall one out of six (14.8%) felt to have been treated roughly like pushed, beaten, slapped, pinched, physically restrained, or gagged. About one third (28.5%) reported to have been shouted, scolded, insulted, threatened, or talked to rudely. For
most women (85.8%) the health professionals did not explain the drugs given, and more than half (55%) didn’t feel involved in decisions about their care, nor were asked for permission or consent before performing procedures (57%). Less than a quarter (21.0%) thought that health professionals took the best of care of them or did everything they could to help control their pain (21.8%).
***Thank you for this comment. We agree with your observation. We have now given more emphasis to this point, citing reference documents related to human rights, as well as the WHO statement on disrespect and abuse during childbirth at facility level.

10. Please explain the following statements further – not clear as is:
• “Interestingly, women’s satisfaction had a very poor correlation with the Bologna score, but a moderate correlation with PCMC, suggesting that women’s satisfaction may have been more affected by the “experience of care” than by the “provision of care”, and that the two domains were very poorly interconnecting, in women’s views.”
***Thank you for the suggestion. We have explained this statement further. Bologna score focuses on “practices” while the PCMC focuses on the “experience “of care. In many settings these two aspect are not at equal quality. This emphasizes that improving good practices (as requested by the evidenced-based movement) without attention to the “relation” is not enough.

• Notably, in this study in Sri Lanka some of items of the Bologna score actually indicated good practices, for example delivery in non-supine position was much more frequent tin this study than what reported in a study in Italy [35].

***We have explained this statement further. Large use of CTG during delivery in Italy may explain this difference.

• On the other side, the PCMC score significantly changed in different ethnic group, in women with more pregnancies, and by type of professionals that assisted the delivery.

***We have explained this statement further.

6. PLOS authors have the option to publish the peer review history of their article ([what does this mean?](https://posta.um.fvg.it/owa/redir.aspx?C=bqFfz4_6b5_TkWygQb7FC_ZtE021mDbzZ_NxbCZeBzPM3BIxE1vYCA..&URL=https%253a%252f%252fjournals.plos.org%252fplosone%252fs%252feditorial-and-peer-review-process%2523loc-peer-review-history)). If published, this will include your full peer review and any attached files.


If you choose “no”, your identity will remain anonymous but your review may still be made public.


**Do you want your identity to be public for this peer review?** For information about this choice, including consent withdrawal, please see our [Privacy Policy](https://posta.um.fvg.it/owa/redir.aspx?C=zyXlYQCLpTwZaCiUmKDBucj1sv0rjTxp9lQqw3GIn6DM3BIxE1vYCA..&URL=https%253a%252f%252fwww.plos.org%252fprivacy-policy).

Reviewer #1: No

Reviewer #2: **Yes:** Saraswathi Vedam

[NOTE: If reviewer comments were submitted as an attachment file, they will be attached to this email and accessible via the submission site. Please log into your account, locate the manuscript record, and check for the action link "View Attachments". If this link does not appear, there are no attachment files.]

While revising your submission, please upload your figure files to the Preflight Analysis and Conversion Engine (PACE) digital diagnostic tool, [https://pacev2.apexcovantage.com/](https://posta.um.fvg.it/owa/redir.aspx?C=D4YjtreJtY9wHKrF7b2-hVzO9dUDO4Z72I2PWzAzQHrM3BIxE1vYCA..&URL=https%253a%252f%252fpacev2.apexcovantage.com%252f). PACE helps ensure that figures meet PLOS requirements. To use PACE, you must first register as a user. Registration is free. Then, login and navigate to the UPLOAD tab, where you will find detailed instructions on how to use the tool. If you encounter any issues or have any questions when using PACE, please email PLOS at [figures@plos.org](https://posta.um.fvg.it/owa/redir.aspx?C=xzGmalPOFwDVV2F5ftvOSzi2iupuWsIUCBrSJhBlIezM3BIxE1vYCA..&URL=mailto%253afigures%2540plos.org). Please note that Supporting Information files do not need this step.
