# Supplementary material for: Correlation among experience of person-centered maternity care, provision of care and women’s satisfaction: Cross sectional study in Colombo, Sri Lanka
Source: PLoS One. 2021 Apr 8;16(4):e0249265. doi: 10.1371/journal.pone.0249265 (PMC8031099; doi:10.1371/journal.pone.0249265)
Supplement: Attachment — Submitted filename: Rebuttal Letter 0303.docx [file pone.0249265.s011.docx]

PONE-D-20-18688R1
Correlation among experience of person-centered maternity care, provision of care and women’s satisfaction: cross sectional study in Colombo, Sri Lanka
PLOS ONE


Reviewers' comments:

Reviewer's Responses to Questions

**Comments to the Author**

1. If the authors have adequately addressed your comments raised in a previous round of review and you feel that this manuscript is now acceptable for publication, you may indicate that here to bypass the “Comments to the Author” section, enter your conflict of interest statement in the “Confidential to Editor” section, and submit your "Accept" recommendation.

Reviewer #1: All comments have been addressed

Reviewer #2: (No Response)

2. Is the manuscript technically sound, and do the data support the conclusions?

The manuscript must describe a technically sound piece of scientific research with data that supports the conclusions. Experiments must have been conducted rigorously, with appropriate controls, replication, and sample sizes. The conclusions must be drawn appropriately based on the data presented.

Reviewer #1: Yes

Reviewer #2: Yes

3. Has the statistical analysis been performed appropriately and rigorously?

Reviewer #1: Yes

Reviewer #2: Yes

4. Have the authors made all data underlying the findings in their manuscript fully available?

The [PLOS Data policy](https://posta.um.fvg.it/owa/redir.aspx?C=NunvFtiVGzy12pWzE89I5xff-E7wA89YU4o6qTpqbmfCcqWT6NfYCA..&URL=http%253a%252f%252fwww.plosone.org%252fstatic%252fpolicies.action%2523sharing) requires authors to make all data underlying the findings described in their manuscript fully available without restriction, with rare exception (please refer to the Data Availability Statement in the manuscript PDF file). The data should be provided as part of the manuscript or its supporting information, or deposited to a public repository. For example, in addition to summary statistics, the data points behind means, medians and variance measures should be available. If there are restrictions on publicly sharing data—e.g. participant privacy or use of data from a third party—those must be specified.

Reviewer #1: Yes

Reviewer #2: Yes

5. Is the manuscript presented in an intelligible fashion and written in standard English?

PLOS ONE does not copyedit accepted manuscripts, so the language in submitted articles must be clear, correct, and unambiguous. Any typographical or grammatical errors should be corrected at revision, so please note any specific errors here.

Reviewer #1: Yes

Reviewer #2: Yes

6. Review Comments to the Author

Please use the space provided to explain your answers to the questions above. You may also include additional comments for the author, including concerns about dual publication, research ethics, or publication ethics. (Please upload your review as an attachment if it exceeds 20,000 characters)

**Reviewer #1:**

1) Thank you for your revision. The revised analyses and substantial discussion have greatly improved the paper. I especially appreciated the more detailed discussion surrounding women’s perceptions and normalization of disrespectful treatment.

*** Many thanks you for your appreciation

2) You addressed some limitations with satisfaction as a measure, but there are other biases and limitations that should be addressed regarding satisfaction. For example, using satisfaction as a dichotomous variable, splitting satisfaction at 6 and above, will pose problems especially because respondents will often select the mid-point option as a cognitive bias. It appears that this is bias was also present in your sample, since over 15% of your sample selected 5. This suggests that a large fraction of those who selected the middle option were subject to cognitive biases and not influenced by other risk factors and likely overestimates the proportion of those who were “not satisfied.”
   Rodway, P., Schepman, A., and Lambert, J. (2012). Preferring the One in the Middle: Further Evidence for the Centre-stage Effect. Applied Cognitive Psychology, 26 (2), 215-222 DOI: 10.1002/acp.1812

**Thank you for the interesting reference provided. Satisfaction was collected on a liker scale (1 to 10). However, when looking at the distribution of the satisfaction scores, Women’s overall satisfaction was not normally distributed (Shapiro-Wilk p<0.001) as shown in Fig1d. The median satisfaction score was 7 (IQR range: 5 to 9) with 295 women (73.7%) above the minimum satisfaction limit of 6; 186 (46.5%) had a satisfaction score between 6-8, and 109 (27.3%) a satisfaction score of >8 out of 10. We believe that this distribution does not suggest a cognitive bias. In addition, data on satisfaction had a good correlation with the PCMC scores (Spearman r= 0.58, p<0.001) (Fig 4C), with mothers who had a bad experience of PCMC expressing low satisfaction, and mothers with a good experience of PCMC reporting higher satisfaction, and thus suggesting that mothers genuinely expressed their satisfaction, based on their experience of care.

3) Your discussion could also benefit by addressing limitations of the Bologna score. I am not sure that it can be considered a measure of “the provision of care” as defined by the WHO QOC framework for maternal and newborn health. Perhaps it might be better described as “recommended clinical practices” or something that highlights clinical practices. Both companion of choice and delivery in the position of choice are considered to be respectful care practices and I believe are classified under the “experience of care.” Because of this, I do not think the Bologna score can be considered an appropriate measure of the “provision of care.”
   Additionally, it should be mentioned that the correlation between PCMC and Bologna care should be interpreted conservatively, since it may be partially due to overlapping items. For example, the PCMC scale has items relating to companionship during labor and delivery, and whether the woman was able to deliver in the position she wanted.

*** Many thanks for your thoughtful comment. The Bologna score include 5 indicators: 1) presence of a companion at the time of birth; 2) use of partograph; 3) absence of labor stimulation (use of oxytocin, external pressure of the uterine fundus, or episiotomy); 4) delivery in non-supine position; 5) skin-to-skin contact with their newborn immediately post-partum. You are very much right in saying that the first of this indicator is considered by the WHO framework as indicator of “experience of care”. However, all the other four are categorized under “provision of care”. In addition, labor companion and non-supine position at birth are known to also have an impact on outcomes such as the incidence of operative delivery, length of labor etc. For this reason, we generalized the Bologna score as a tool to assess primarily provision of care. We have made this clear in the paper now.

We also would like to add that indictors of QOC is a complex construct which involves many overlapping parameters other than evidence-based practices alone. Generally, tools that are used to measure provision of care do not guarantee that they measure the provision of care alone, and many aspects of QOC have both component.

We reported in the paper that the correlation in between Bologna and PCMC was very low (Pearson r=0.20, p<0.001). As you rightly pointed out, the PCMC include, out of 30 total items, the two items: “**Were you** **allowed** to have someone you wanted (outside of staff at the facility, such as family or friends) to stay with you during labour?” and “During the delivery, **do you feel like you were able** to be in the position of your choice? “These two items indeed refer to two items of the Bologna Score, but they explore the point from the angle of the experience of the mothers (ie. **Were you** **allowed?** **do you feel like you were able** ‘). This is a very different perspective/angle/lens from the one explored in the Bologna, which is merely factual.

4) Minor points:

- Please address misspelled terms in footnotes of Table 1: “caesarian” “nera” “lapartotomy” *** This has been corrected
- Line 304: “One third (28.5%)…” It seems that ‘over one-quarter’ or ‘nearly one-third’ would be more accurate than ‘one-third.’ *** This has been corrected
- Line 454: “heart” is misspelled *** This has been corrected
- The figures are quite blurry. I am not sure if this because the images are of low resolution or if the system has distorted images. *** This is due to the low resolution requested in the submission. We will provide higfh resolution images once the paper has been accepted
- Figure 3, please correct “Skin-Skin” *** This has been corrected
- Figure 4, especially because some of your graphs include plots using only discrete values (i.e. 4b), please consider illustrating density in your plots (for example, using “jitter” or dodging points). *** Ilaria

**Reviewer #2:**

1) Thank you for the opportunity to read this revised version. The paper is greatly improved in clarity and impact as a result of the edits and additions made in response to reviewers’ comments.

*** Many thanks you for your appreciation

2) My remaining suggestions are mostly very minor copyediting recommendations; however, I do have one important note to the authors and editor: This study adds to the growing body of literature that shows mistreatment and human rights violations during childbearing is a global and widespread phenomena (1/6), and is even more prevalent among historically disenfranchised communities (2/3). It is an important contribution to our understanding not only frequency of disrespect and abuse, but that these person-centred metrics of quality care are important outcome in their own right (eg. They do not need to be linked to other maternal newborn adverse outcomes, or patient satisfaction to merit urgent attention). Yet, the authors appear to be hesitant to let the data speak to this mandate, reserving the emphasis in the discussion solely to the key metric they chose to use: patient satisfaction. Perhaps they could at least state more clearly in their conclusions (abstract and paper) that “findings indicate evidence of poor quality care across several domains of mistreatment in childbirth in Sri Lanka. [and] Patient satisfaction as an indicator of quality care is inadequate to inform health systems reform”. I can understand a reluctance to speak of prevalence of the most adverse outcomes (mistreatment) based on a sample size of 400 in the region (authors frame this analysis as pilot data), and I agree that discussions generally focus on findings per the originally intended methods. However, when results show egregious harm, in any study, it seems that researchers are obligated to name and elevate these findings and the urgency of rectifying harm and future targeted research on these matters. This is especially true when the findings align with incidence reported other published literature on the subject as cited.

*** Thank you for your comments. We sincerely appreciate this suggestion, and we have now revised the abstract and discussion.

Our findings indicate and reflect that the quality of care is poor across several domains of mistreatment during child birth in Sri Lanka. Patient satisfaction scores alone are inadequate to inform health systems reforms.

Local authors have undertaken a series of focus group discussions to better examine women’s views (these data will be published in a separate publication). Based on data collected, a multifaceted intervention will be hopefully designed.

3) Copy edits and typographical errors
   Line 66 – Keywords - Suggested these edits to maximize searches for this article: Quality care, respectful maternity care, person-centered/person-centred, mistreatment, childbirth, disrespect and abuse (researchers in this field are unlikely to see satisfaction as an important component to include as noted by reviewer comments) *** This has been corrected
   Line 96 (comma instead of full stop) *** This has been corrected
   109-110 missing word: also defined [as] patient-centred, …*** This has been corrected
   Line 142 suggest using “births” instead of deliveries – fast becoming the norm for respectful language (eg women give birth, providers *** This has been corrected
   Line 293-296 The following sentence is unclear – either there were significant differences with some factors having lower scores or the scores were significantly lower for xxxx than xxxx*** This has been corrected
   Additionally, other differences among the
   294 full PCMC score (47.1; 95%CI 45.9-48.2) and “dignity and respect” (57.2; 95%CI 55.8-58.6), and 295 “supportive-care” subscores (50.5; 95%CI 49.0-51.9) were significantly lower (adjusted 296 p≤0.002 for all comparisons). PCMC not rescaled values are reported in Tables S2 and S3. *** This has been corrected
   Line 303 awkward phrasing: 303 six (14.8%) felt to have been treated roughly – perhaps change to one out of six (14.8%) reported that they were treated roughly --or—one out of six (14.8%) reported mistreatment – all of the listed factors constitute mistreatment by global definitions. *** This has been corrected
   Line 305 – best not to start a sentence with a percentage – perhaps rephrase: Most women (85.8%) reported that….*** This has been corrected
   Line 357 women “at” their second pregnancy should be in their second pregnancy women….. or women [ in] their second pregnancy*** This has been corrected
   422 mis-treated should be mistreated *** This has been corrected
4) Other comments
   Line 368 “No factor was associated with women’s satisfaction” (sensitivity analysis just parity and non-supine position) -- this result shows that satisfaction is a poor discriminator of quality care and that finding should be discussed – with recommendation that future studies report on PCMC scores, and incidence of mistreatment as a stand alone indicator of quality, safety, rights.

*** Thank you very much for your inspiring comment. We have added this in the discussion.

5) Line in 394-399 that discusses above is too mild given the findings that 1/6 to ½ experience serious mistreatment – as written the discussion does not address that only one modifiable experience of care factor (birth position) was even moderately correlated – hence satisfaction is not measuring quality or the correlation shou

*** Thank you very much for your inspiring comment. We have added this in the discussion.

6) Lines 472-476 should immediately follow Line 427 as finding on differential experience by ethnicity are also socio-demographic predisposing factors. Then “ethnicity” should be added to the line 431 …-and better document how education, [ethnicity, social class], empowerment and values…..

***Very good suggestion, we have revised the text accordingly
